# IL-17-mediated mitochondrial dysfunction impairs apoptosis in rheumatoid arthritis synovial fibroblasts through activation of autophagy

**DOI:** 10.1038/cddis.2016.490

**Published:** 2017-01-19

**Authors:** Eun Kyung Kim, Jeong-Eun Kwon, Seon-Young Lee, Eun-Jung Lee, Da Som Kim, Su-Jin Moon, Jennifer Lee, Seung-Ki Kwok, Sung-Hwan Park, Mi-La Cho

**Affiliations:** 1 The Rheumatism Research Center, Catholic Research Institute of Medical Science, The Catholic University of Korea, Seoul, South Korea; 2 Laboratory of Immune Network, Catholic Research Institute of Medical Science, The Catholic University of Korea, Seocho-gu, Seoul, South Korea; 3 Divison of Rheumatology, Department of Internal Medicine, The Catholic University of Korea, Seoul, South Korea

## Abstract

Fibroblast-like synoviocytes (FLSs) are a major cell population of the pannus that invades cartilage and bone in rheumatoid arthritis (RA). FLS resistance to apoptosis is a major characteristic of RA. The aims of this study were to investigate the effects of interleukin-17 (IL-17) and IL-17-producing T helper (Th17) cells on resistance to apoptosis in FLSs from RA patients (RA FLSs) and their roles in mitochondrial dysfunction and autophagy. Mitochondrial function was assessed in RA FLSs and FLSs from osteoarthritis patients (OA FLSs). FLSs were treated with IL-17 and their morphological features, respiratory level and mitochondrial gene expression were measured. The effects of IL-17 and Th17 cells on the relationship between autophagy and apoptosis were evaluated by measuring the expression of apoptosis-related genes using sodium nitroprusside or 3-methyladenine. The mitochondria of FLSs isolated from RA and osteoarthritis patients displayed different morphological and physiological features. RA FLSs exhibited greater autophagosome formation and greater dysfunction of mitochondrial respiration compared with OA FLSs. IL-17 induced mitochondrial dysfunction and autophagosome formation in RA FLSs, suggesting that they were resistant to apoptosis. Autophagy-related antiapoptosis induced by IL-17 was restored by inhibition of autophagy, suggesting a relationship between mitochondrial dysfunction and cell survival in RA FLSs. Th17 cells and IL-17 increased autophagy of RA FLSs by causing mitochondrial dysfunction. Our findings suggest that, in RA, interactions between RA FLSs and Th17 cells may be involved in the tumorous growth of FLSs and the formation of pannus in joints.

Rheumatoid arthritis (RA) is the most common autoimmune disease and is characterized by progressive joint destruction and functional disability in affected people. The pathognomonic finding of RA is the expansive synovial tissue, called pannus, which erodes cartilage and bone at the cartilage–bone interface. Fibroblast-like synoviocytes (FLSs) of the synovial lining can produce local inflammatory cytokines and proteolytic enzymes such as matrix metalloproteinases, which degrade the extracellular matrix.^1^ The pannus behaves like a locally invasive tumour, and the potential imbalance between the growth and death of RA FLSs is considered a target in the treatment of the disease.

Accumulating scientific evidence shows that interleukin 17 (IL-17) and IL-17-producing T helper (Th17) cells play critical roles during the development and progression of RA.^2, 3^ Although the exact pathogenesis of RA remains unclear, data from experimental models suggest that IL-17 plays a role in pannus growth,^4^ structural destruction of rheumatoid joints through receptor activator of nuclear factor κB ligand-independent osteoclastogenesis,^5^ and synovial neoangiogenesis.^6^

Mitochondrial dysfunction is emerging as a mechanism underlying various inflammatory and autoimmune diseases including cancer, atherosclerosis, neurodegenerative diseases, diabetes, obesity and autoimmune diseases.^7, 8, 9, 10^ These diseases become worse when accompanied by systemic inflammation and oxidative stress.^11^ Mitochondria, indispensable organelles in mammalian cells, supply adenosine triphosphate (ATP), buffer calcium gradients and deposit unique molecules in cells. The mitochondrial respiratory machinery produces ATP by coupling oxygen and nutrients through oxidative phosphorylation (OxPhos); however, the exposure of cells to oxidative stress leads to mitochondrial damage, which stimulates their eventual elimination through autophagy.^12, 13, 14^

Defective mitochondria are also degraded by autophagy, a process involving lysosomal degradation that restores homeostasis in multicellular organisms. Autophagy and autophagy proteins protect cells from cellular stressors such as viruses or bacteria, and control cell death, immune responses and inflammation.^15, 16, 17, 18^ The two main self-destructive processes, autophagy and apoptosis, are closely related, and their interaction determines cell fate. Most of the processes of stress-related autophagy precede apoptosis, are mediated by caspase 6, caspase 7 and cytochrome c, and lead to the formation of apoptosomes and activated caspase 3.^19, 20^ However, autophagy is known to inhibit indiscriminate cell death to prevent unwarranted removal of functional mitochondria.^21, 22^

Autophagy plays a crucial role in immunity and inflammation.^23^ Excessive production of reactive oxygen species (ROS) by defective mitochondria may initiate inflammation.^24^ Mitochondrial oxidative stress caused by infection or inflammatory disease increases the secretion of chemokines and inflammatory cytokines, which stimulate infiltration by lymphocytes such as T helper (Th) cells.^25^ Mutations in mitochondrial genes are related to the pathogenesis and local inflammation involved in RA.^26, 27^

We hypothesized that another pathological mechanism of mitochondrial dysfunction in Th17 cells is involved in RA. In the present study, we examined whether IL-17 can intensify RA severity by causing mitochondrial dysfunction. To test whether inflammation-related mitochondrial dysfunction is involved in RA, we studied FLSs isolated from patients with osteoarthritis (OA) or RA. We evaluated dysfunction of mitochondrial respiration, autophagy and autophagy-related apoptosis induced by IL-17 in these FLSs to examine further the relationship between inflammation, mitochondria and cell death in RA. All of our findings suggest that Th17 cells and IL-17 accelerate the autophagy-mediated antiapoptotic process in FLSs and contribute to the pathology of RA.

## Results

### Mitochondrial dysfunction is increased in RA FLSs

**Figure 1:**
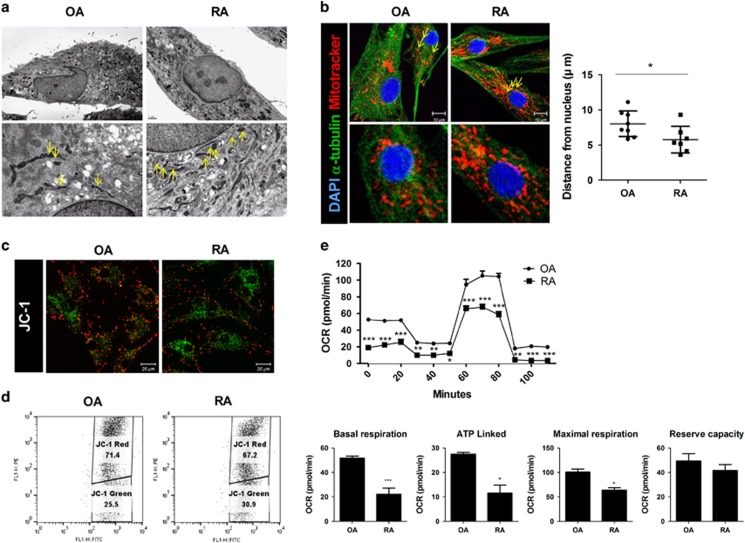
Mitochondrial dysfunction increases in RA FLSs. (**a**) Transmission electron microscopy images of FLSs from OA and RA patients. Arrowheads indicate mitochondria. Scale: 2 μm (upper panels), 0.5 μm (lower panels). (**b**) OA and RA FLSs were immunostained with MitoTracker Red CMXROS (red), anti-α-tubulin (green), and DAPI (nuclei, blue). quantification of perinuclear mitochondrial distance in confocal micrographs of OA and RA FLSs. Data represent means±S.D. of five different culture experiments. The mitochondrial membrane potential was measured using JC-1 dye. (**c**) Confocal microscopy and (**d**) flow cytometry analysis. A representative density plot shows the OA and RA FLSs. (**e**) OCR in OA and RA FLSs (data are representative of three experiments). Quantification of mitochondrial respiration in OA and RA FLSs. The data are expressed as the mean±S.D. (or S.E.M.). *P<0.05, **P<0.01, ***P<0.001

To investigate the mitochondrial dysfunction of RA FLSs, the morphologic changes of mitochondria were investigated in RA and OA FLSs isolated from synovia. Electron microscopy showed abnormal cristae in mitochondria and autophagosomes in both OA and RA FLSs. Interestingly, abnormal dark cristae in mitochondria were noted more frequently in RA FLSs compared with OA FLSs, which suggested a more abnormal mitochondrial function of RA FLSs^28^ (Figure 1a). MitoTracker staining showed significantly greater fragmentation and perinuclear clustering of mitochondria in RA FLSs compared with OA FLSs (Figure 1b). The distance of mitochondria from nuclei was shorter in RA FLSs than in OA FLSs.

These morphological differences observed in RA FLSs may imply mitochondrial dysfunction. Therefore, we next studied the functional impairment in mitochondria of RA FLSs. Confocal microscopy and flow cytometric analysis showed a significantly increased ratio of green to red staining in RA FLSs compared with OA FLSs, which indicated a lower mitochondrial membrane potential in RA FLSs (Figures 1c and d). The respiratory ability of mitochondria was also significantly lower in RA FLSs compared with that in OA FLSs (Figure 1e, upper panel). The basal, maximum and ATP-linked mitochondrial respiratory rates were all lower in RA FLSs than in OA FLSs (Figure 1e, lower panel).

### Pathogenesis of RA is closely related to IL-17

**Figure 2:**
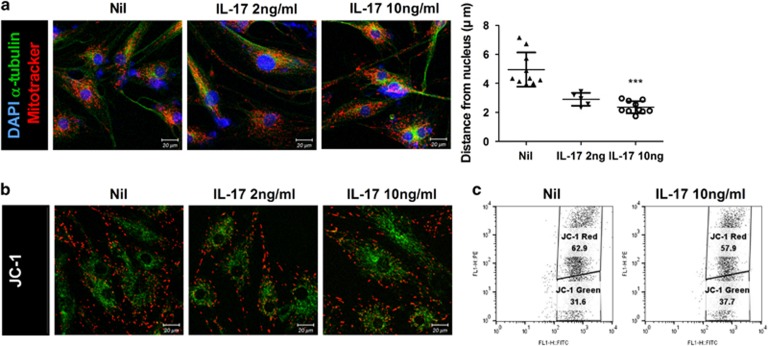
Pathogenesis of RA is mediated by IL-17. FLSs were cultured with IL-17 at concentrations of 0, 2 or 10 ng/ml for 12 h (*n*=5). (**a**) FLSs were immunostained with MitoTracker Red CMXROS (red), anti-α-tubulin (green) and DAPI (nuclei, blue), and the perinuclear mitochondrial distance was quantified in confocal micrographs. A representative density plot shows the RA FLSs. Data represent the mean±S.D. ***P<0.001. Mitochondrial membrane potential was measured using JC-1 dye for confocal microscopy (**b**) or flow cytometry analysis (**c**)

Because IL-17-expressing effector T cells, called Th17 cells, are strongly implicated in RA pathogenesis,^4, 29^ we studied the effects of IL-17 on mitochondrial function in RA FLSs. RA FLSs were incubated with IL-17 at a concentration of 2 or 10 ng/ml. MitoTracker staining showed that IL-17 treatment intensified the perinuclear clustering of mitochondria in RA FLSs (Figure 2a, left panel). The distance of mitochondria from nuclei was decreased by IL-17 treatment in a dose-dependent manner (Figure 2a, right panel). The mitochondrial membrane potential was attenuated by IL-17 in RA FLSs (Figures 2b and c). Taken together, these findings suggest that IL-17 may be a critical pathological cytokine that impairs mitochondrial function in RA FLSs.

### IL-17 disrupts mitochondrial respiration in RA FLSs

**Figure 3:**
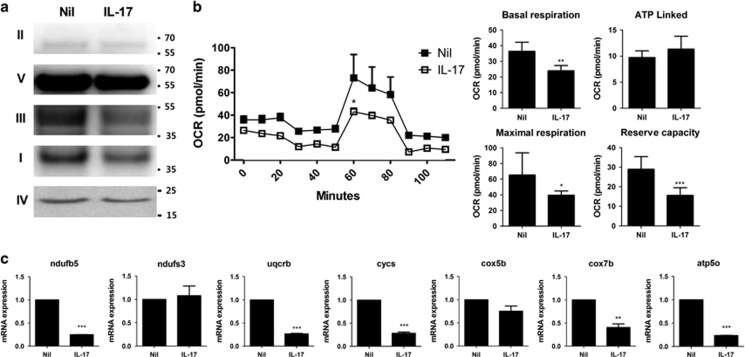
IL-17 disrupts mitochondrial respiration in RA FLSs. (**a**) The expression of OxPhos complex subunits in FLSs was analysed by western blotting. A representative figure is shown. (**b**) OCR in FLSs in the presence or absence of IL-17 (10 ng/ml). The data are representative of three independent experiments. Quantification of mitochondrial respiration in FLSs. (**c**) The expression level of mitochondrial OxPhos genes were determined using RT-qPCR in FLS incubated in the presence or absence of IL-17 (10 ng/ml) for 12 h. The data are expressed as the mean±S.D. (or S.E.M.). *P<0.05, **P<0.01, ***P<0.001

The main role of mitochondria is to produce ATP through respiration. We next examined whether IL-17 can cause changes in the composition or level of the respiratory machinery. Among the OxPhos complexes studied, the expression of complexes I and III was inhibited by IL-17 (Figure 3a). The basal and maximum respiratory levels, and reserve capacity in mitochondria, were also markedly reduced by IL-17 (Figure 3b). These results suggested that each component of the respiratory apparatus was affected and contributed to the attenuation in mitochondrial respiratory function. Surprisingly, reverse transcription-quantitative polymerase chain reaction (RT-qPCR) results showed that IL-17 decreased the expression of the ATP- or respiration-related gene *NDUFB5*, a component of the respiration complex I; *UQCRB*, *CYCS*, *COX5B* and *COX7B*, components of respiration complex III; and *ATP5O*, a component of the F-type ATPase found in the mitochondrial matrix, in RA FLSs (Figure 3c). These data suggest that IL-17 decreases the expression of the genes for OxPhos complex components and that this may lead to impaired respiratory capacity in mitochondria of RA FLSs.

**Figure 4:**
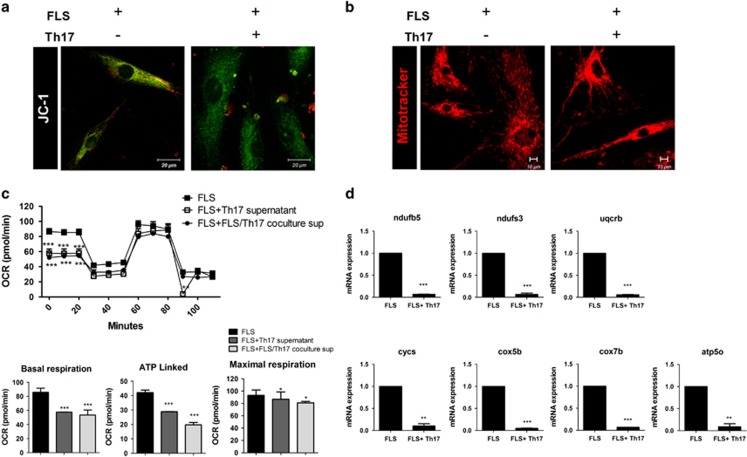
Th17 disrupts mitochondrial respiration in RA FLSs. FLSs isolated from RA patients were co-cultured with or without Th17 cells for 12 h. (**a**) The mitochondrial membrane potential was measured using JC-1 dye and confocal microscopy. Green indicates decreased membrane potential. (**b**) Mitochondria were immunostained with MitoTracker Red CMXROS (red) and confocal microscopy images were captured. (**c**) The OCR was measured in FLSs co-cultured with or without supernatant collected from Th17 cells or from Th17 cells that had been co-cultured with FLS. Data are representative of three experiments. Quantification of mitochondrial respiration in RA FLSs. (**d**) Relative gene expression associated with mitochondrial OxPhos in RA FLSs. The data are expressed as the mean±S.D. (or S.E.M.). *P<0.05, **P<0.01, ***P<0.001

Next, we tried to identify the direct effects of Th17 cells on mitochondria of RA FLSs. RA FLSs were co-cultured with or without *in vitro*-differentiated human Th17 cells. Confocal microscopy showed that RA FLSs co-cultured with Th17 cells had a lower mitochondrial membrane potential compared with untreated cells (Figure 4a). Perinuclear clustering of mitochondria in RA FLSs increased after co-culture with Th17 cells (Figure 4b). The reserve respiratory capacity was not affected by co-culture with supernatant collected from Th17 cell or Th17 cells that had been co-cultured with FLS; however, the basal respiratory level, ATP-linked respiration and maximum respiratory level decreased markedly after co-culture with supernatant collected from Th17 cells or Th17 cells co-cultured with FLS (Figure 4c). The mRNA levels of OxPhos components were also decreased after co-culture of RA FLSs with Th17 cells (Figure 4d). This indicated that Th17 cells induced mitochondrial respiratory dysfunction in RA FLSs. Taken together, these data suggest that Th17 cells and the proinflammatory cytokine IL-17 cause both mitochondrial malformation and functional impairment of respiratory capacity and ATP production. We speculate that IL-17 may play a critical role in mitochondrial dysfunction in RA FLSs.

### IL-17 stimulates autophagy by inducing mitochondrial dysfunction in RA FLSs

**Figure 5:**
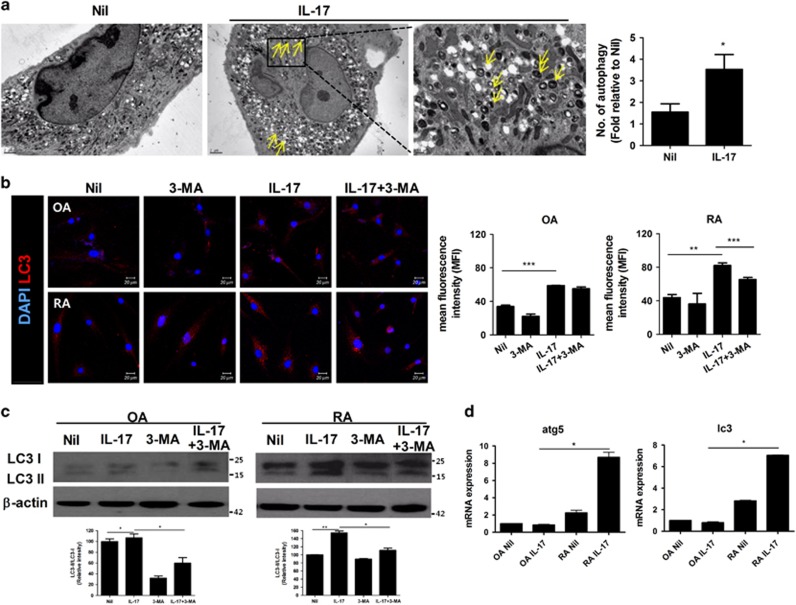
IL-17 induces autophagy by causing mitochondrial dysfunction in RA FLSs. (**a**) RA FLSs were cultured in the presence or absence of IL-17 (10 ng/ml) and transmission electron microscopy was performed. Arrowheads indicate autophagosomes. The right panels show magnified autophagosomes in RA FLSs cultured in the presence of IL-17. The number of autophagosomes in FLSs was quantified on the electron microscopy images. The data in the graph on the right are expressed as mean±S.D. of five different experiments. (**b**) OA FLSs and RA FLSs were immunostained for the autophagosome marker with anti-LC3 antibody and analysed by confocal microscopy. Scale: 20 μm. (**c**) Expression of LC3 in RA FLSs cultured with 3-MA (1 mM) in the presence or absence of IL-17 (10 ng/ml) was analysed by Western blotting. The LC3-II/LC3-I ratio was quantified. (**d**) The gene expression of *ATG5* or *LC3* associated with autophagy in FLSs cultured in the presence or absence of IL-17 (10 ng/ml) was quantified using RT-qPCR. The data are expressed as the mean±S.D. *P<0.05, **P<0.01, ***P<0.001

Damage caused by stressors such as ROS increases mitochondrial autophagy (also termed mitophagy).^30^ To study further the abovementioned results showing that IL-17 and Th17 cells cause mitochondrial dysfunction, we next examined the influence of IL-17 on autophagy in RA FLSs. FLSs were treated with or without IL-17 and autophagy was assessed. Electron microscopy showed that IL-17 treatment doubled the number of autophagosomes with dark cristae in RA FLSs (Figure 5a). This increase in autophagy was confirmed by the IL-17-induced increase in staining for LC3, a marker of autophagy, in OA FLSs and RA FLSs. In addition, 3-methyladenine (3-MA), which inhibits autophagosome formation by inhibiting phosphoinositide 3-kinase, reduced LC3 expression (Figure 5b). Western blot analysis showed that the LC3-II/LC3-I protein ratio increased from 0.94 in untreated cells to 1.12 after IL-17 treatment of RA FLSs. However, there was little change in this ratio in OA FLSs stimulated with IL-17. The 3-MA-treated FLSs showed decreased expression of LC3 compared with IL-17- or IL-17+3-MA-treated FLSs (Figure 5c). We measured the mRNA levels of autophagy markers including *ATG5* and *LC3* in OA FLSs and RA FLSs. IL-17 increased the expression levels of *ATG5* and *LC3* in RA FLSs but caused few effects on the expression of these markers in OA FLSs (Figure 5d). Our data suggest that IL-17 can induce mitochondrial dysfunction, which results in autophagy and the abnormal expression of autophagy-related genes in RA FLSs. In other words, IL-17-mediated inflammation seems to stimulate autophagy, which leads to mitochondrial dysfunction in RA FLSs.

### Antiapoptosis is increased in RA FLSs by induction of IL-17-mediated autophagy

**Figure 6:**
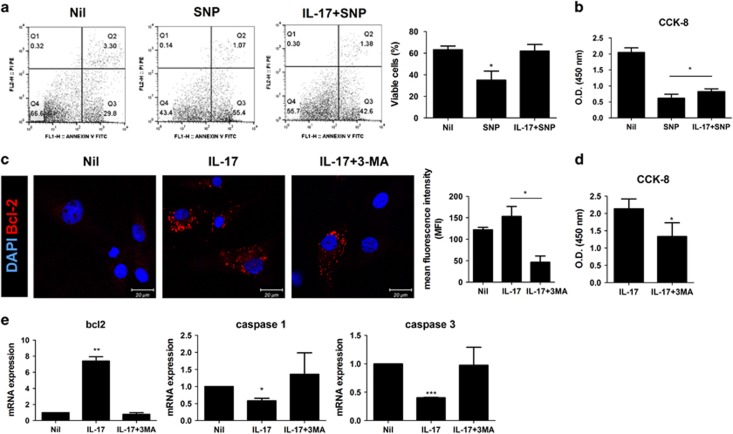
Induction of IL-17-mediated autophagy increases antiapoptosis in RA FLSs. (**a**) Apoptosis was induced by treatment of FLSs with 1 mM SNP. The degree of apoptosis was assessed by flow cytometry using propidium iodide and Annexin V staining. (**c**) Inhibition of autophagy with 3-MA decreased the expression of BCL-2 in RA FLSs in the presence of IL-17 (10 ng/ml). FLSs from RA were immunostained using anti-BCL2 antibody as the antiapoptosis marker and analysed by confocal microscopy. Scale: 20 μm. (**b**, **d**) The viability of synoviocytes were determined using a CCK-8 assay kit. (**e**) RT-qPCR was performed to quantify the relative expression of genes associated with apoptosis or antiapoptosis in RA FLSs cultured in the presence or absence of IL-17 (10 ng/ml) for 12 h. The data are expressed as the mean±S.D. *P<0.05, **P<0.01, ***P<0.001

Autophagy is related closely to cell death because it induces apoptosis or necrosis-related proteins.^18^ We next evaluated the effects of IL-17 on cell death in FLSs. As seen in Figure 6a, flow cytometric analysis showed that sodium nitroprusside (SNP) increased apoptosis-mediated cell death and reduced cell viability from 66.6% to 43.4%. By contrast, IL-17 treatment restored cell viability to 55.7%. The CCK-8 cell viability assay also showed that IL-17 protected cells from apoptosis caused by SNP and fully restored cell viability (Figure 6b). Immunocytochemical staining indicated that IL-17 treatment increased the expression of the antiapoptotic protein BCL2 in RA FLS mitochondria (Figure 6c). However, IL-17 was unable to reverse the effects on cell death induced by 3-MA, which inhibits autophagosome formation by inhibiting phosphoinositide 3-kinase (Figure 6d). This suggests that the antiapoptotic function of IL-17 depends on autophagy.

Next, we tested whether the expression of apoptosis-related genes is dependent on IL-17 or autophagy. We performed RT-qPCR to examine the expression of genes for regulatory molecules of apoptosis such as BCL2 or caspase. The expression of the antiapoptotic protein BCL2 was increased sevenfold by IL-17 treatment compared with the control. The expression of proapoptotic molecules such as caspase 1 and caspase 3 were reduced by IL-17 treatment (Figure 6e). Co-treatment with 3-MA and IL-17 reversed the effects of IL-17 on the expression of BCL2 and increased the expression of caspase 1 and caspase 3. Cytochrome c expression was also decreased by IL-17 and was recovered by 3-MA treatment (data not shown). These data indicate that IL-17 is a potent inhibitor of apoptosis by induction of autophagy in RA FLSs.

## Discussion

In the present study, we investigated the possible mechanisms responsible for the effects of Th17 cells and IL-17 on mitochondrial dysfunction in RA FLSs. Although Th17 cells and IL-17 are known to be critical players during the RA inflammatory process,^29, 31, 32^ ours is the first study to show an association between IL-17- and Th17 cell-induced accelerated mitochondrial dysfunction and consequent induction of autophagy in RA FLSs. Our data suggest that mitochondrial malfunction in arthritis can be induced or augmented by IL-17 and/or Th17 cells.

We compared the functional differences in mitochondria between RA and OA FLSs. Although the destructive physiology of RA and OA is similar, the functions of mitochondria differ between these two diseases. The hallmarks of mitochondrial dysfunction, such as perinuclear clustering of mitochondria, abnormal dark cristae, autophagosome formation, and decreased membrane potential and respiration indicated that the inflammatory environment makes FLSs more vulnerable to mitochondrial malfunction. We also found that the distance of mitochondria from nuclei was shorter, which implies mitochondrial dysfunction, in RA FLSs than in OA FLSs (Figure 1a). Such asymmetric mitochondrial distribution may impair the capacity for ATP generation.^33, 34^ This finding was confirmed by the lower respiration and membrane potential of mitochondria in RA FLSs (Figures 1c–e). The effects of inflammatory Th17 cells and IL-17 were more evident in RA FLSs. Treatment with IL-17, the main cause of RA, intensified perinuclear clustering of mitochondria and decreased the membrane potential (Figure 2), a finding that was similar to the findings shown in Figure 1. Taken together, our data suggest that the FLSs accompanying inflammation in RA are more susceptible to attack by Th17 cells.

Morphological and physiological disorders of mitochondria reflect mitochondrial dysfunction and imply changes in the expression of components of the respiratory machinery. IL-17 and Th17 cells decreased the mitochondrial respiratory level and the protein levels of components of complex I, the largest complex in the respiratory chain, respiration complex III in the mitochondrial inner membrane and ATPase type-F component in the matrix (Figures 3 and 4). In our data, the effects of IL-17 were so significant that they disabled the main function of mitochondria, energy production by respiration. These destructive functions of IL-17 are related to the formation of autophagosomes. IL-17 treatment increased the expression of autophagy-specific markers such as LC3-I, LC3-II and ATG5 in RA FLSs, which also supports the idea that IL-17 plays a unique role in the differences between RA and OA (Figure 5).

In addition to causing malfunction of mitochondria, IL-17 or co-culture with Th17 cells also inhibited cell death. These effects were reversed by SNP, which increased apoptosis by affecting the expression of antiapoptotic or proapoptotic genes (Figures 6a and b). The expression of antiapoptotic genes such as *BCL2* was increased markedly by IL-17, as we have previously reported.^35^ However, this antiapoptotic function of IL-17 was suppressed by inhibition of autophagy (Figures 6c and d). Co-treatment of FLSs with 3-MA and IL-17 showed clearly that antiapoptotic molecules and proapoptotic molecules are precisely regulated by autophagy and IL-17. We propose that autophagy is related to the mitochondrial malfunction caused by IL-17. In other words, inflammation-related RA is susceptible to the effects of inflammation, inflammation increases autophagy-related gene expression and IL-17 accelerates these pathological mechanisms. Differentiated FLSs increase their deposition of collagen, resulting in synovial fibrosis.^36^ Therefore, the promotion of FLS survival by IL-17 may contribute to differentiation of FLSs and consequently, to synovial fibrosis.

Autophagy-related proteins beclin-1, ATG5 and LC3 are key players in the survival of FLSs in the early phase of RA or OA.^37^ However, FLSs undergo different cell fates in the late phases of RA and OA. In the early stage of bone destruction, beclin-1, LC3-II, ULK1 and ATG5 increase in both RA and OA FLSs, whereas their expression decreases in the late phase of OA.^38, 39^ Increased expression of beclin-1 and LC3 inhibits cell death in RA FLSs by stimulating autophagy.^40^ Antiapoptotic factors and autophagy increase, and proapoptotic factors decrease, which results in the survival of RA FLSs.^40, 41, 42, 43^ By contrast, continued mechanical stress may decrease the abundance of ULK1, beclin-1 and LC3, which leads to cell death of chondrocytes in OA FLSs.^44^ However, IL-17 influences the expression of beclin-1 and promotes autophagy formation in B cells,^45^ which implies that IL-17 is important to the activation of autophagy in RA FLSs. Collectively, our findings suggest that an increased IL-17 level maintains autophagy, which allows the survival of RA FLSs. In other words, IL-17-mediated inflammation contributes to mitochondrial dysfunction and the resultant autophagy in RA FLSs, which subsequently stimulate the infiltration of inflammatory lymphocytic cells including Th17 cells.

Therefore, we suggest that a complex relationship exists between mitochondrial dysfunction, cell survival and IL-17-induced autophagy in RA FLSs. This cooperation between FLSs and Th17 cells in RA seems to create a vicious positive feedback cycle of inflammation and an environment for survival of FLSs. We propose that collaboration between FLSs and Th17 cells may be one mechanism responsible for the tumorous growth of FLSs and formation of pannus in joints in RA.

## Materials and methods

### Isolation and culture of human FLSs

Synoviocytes were isolated by enzymatic digestion of synovial tissue specimens obtained from patients with RA or OA undergoing total joint replacement surgery. The tissue samples were minced into 2- to 3-mm pieces and treated for 4 h with 4 mg/ml of type I collagenase (Sigma-Aldrich, St. Louis, MO, USA) in Dulbecco's modified Eagle's medium (DMEM) at 37 °C in 5% CO_2_. Dissociated cells were centrifuged at 500 g, resuspended in DMEM supplemented with 10% fetal bovine serum, 2 Mm L-glutamine, 100 units/ml of penicillin, and 100 ng/ml streptomycin, and plated in 75-cm^2^ flasks. After overnight culture, floating cells were removed, and the adherent cells were cultivated in DMEM supplemented with 10% fetal bovine serum. The cultures were kept at 37 °C in 5% CO_2_, and the medium was replaced every 3 days. The FLSs were passaged 3–8 times using trypsin–ethylenediaminetetraacetic acid (Gibco, Grand Island, NY, USA). The cells were seeded in six- or 24-well plates, eight-well chamber slides, or 100-mm culture dishes in 10% fetal bovine serum-supplemented DMEM, and then cultured for 12 h at 37 °C. FLSs were stained with allophycocyanin-conjugated anti-CD90 (eBioscience, San Diego, CA, USA) antibody as a fibroblast marker and analysed by flow cytometry. Informed consent was obtained from all participating subjects. The study received approval from the Institutional Review Board for Human Research, Seoul St. Mary's Hospital (KC14TISI0535).

### Isolation and differentiation of peripheral blood mononuclear cells (PBMCs)

Human PBMCs were isolated from heparinized blood samples using Ficoll–Hypaque (GE Healthcare Bioscience, Uppsala, Sweden) density gradient centrifugation. The isolated cells were cultured in RPMI1640 medium supplemented with 10% fetal bovine serum. To purify CD4+ T cells, PBMCs were incubated with CD4-coated magnetic beads and isolated using magnetic-activated cell sorting separation columns (Miltenyi Biotec, Bergisch Gladbach, Germany). Positively selected CD4+ T cells were stimulated with plate-bound anti-CD3 (0.5 μg/ml), soluble anti-CD28 (1 μg/ml; both from BD Biosciences, Sparks, MD, USA), anti-interferon-γ (5 μg/ml), anti-IL-4 (5 μg/ml), recombinant interleukin 1β (IL-1β) (10 ng/ml) and recombinant IL-6 (20 ng/ml) (all from R&D Systems, Minneapolis, MN, USA) for 3 days to achieve polarization of Th17 cells.

### Co-culture of Th17 cells with RA FLSs

RA FLSs were seeded in 24-well plates at 1 × 10^6^/well in 10% DMEM. One day later, the cell culture medium was removed and the cells were cultured in 0.1% insulin–transferrin–selenium-A (ITSA)–DMEM medium, then Th17 cells were layered onto the RA FLSs. Culture plates were incubated at 37 °C for 24 h, and the supernatants were collected.

### Electron microscopy

Cells were fixed in 4% paraformaldehyde and 2.5% glutaraldehyde in 0.1 M phosphate buffer overnight at 4 °C. The cells were washed in 0.1 M phosphate buffer, postfixed with 1% osmium tetroxide for 1 h at 4 °C, dehydrated through graded ethyl alcohol solutions, exchanged through acetone, and then embedded in Epon 812. Ultrathin sections (70–80 nm) were obtained on an ultramicrotome (Leica Ultracut, Leica, Vienna, Austria) and stained with uranyl acetate and lead citrate. Images were acquired at 60 kV on a transmission electron microscope (JEM 1010, JEOL, Tokyo, Japan). The number of autophagosomes was counted manually. For each group, cells were randomly selected for the average number of autophagosomes/cell and the fold change in autophagosomes relative to the untreated control was plotted.

### Analysis of mitochondrial membrane potential

The mitochondrial membrane potential was measured using JC-1 staining, confocal microscopy and flow cytometry. For JC-1 staining for confocal microscopy, 1 × 10^4^ cells were cultured in 0.1% ITSA–DMEM medium in eight-well chamber slides in the presence or absence of IL-17 (10 ng/ml) for 12 h. The cells were washed, the medium was changed to PBS, JC-1 dye was added, and the cells were incubated for 15 min at 37 °C. JC-1-labelled cells were washed in PBS and images were acquired on a confocal laser scanning microscope (LSM 510 Meta; Zeiss, Gottingen, Germany). For flow cytometric analysis of JC-1-stained cells, another 8 × 10^4^ cells were cultured in six-well plates in the presence or absence of IL-17 (10 ng/ml) for 12 h. The cells were harvested, JC-1 dye was added, and the cells were incubated for 15 min at 37 °C, washed, and analysed by flow cytometry on a FACSCalibur instrument (BD Biosciences). The fluorescence of the cells in each well was detected using 485-nm excitation wavelength and 530-nm or 595-nm emission wavelength.

### MitoTracker staining and confocal laser scanning microscopy

For analysis of mitochondria using MitoTracker Red CMXROS (Molecular Probes, Eugene, OR, USA), the cells were seeded in eight-well chamber slides at a density of 1 × 10^4^ cells/well in the presence or absence of IL-17 (10 ng/ml) for 12 h. Mitochondria were stained with the MitoTracker Red probe for 30 min at 37 °C, after which the cells were washed with PBS, fixed with methanol, permeabilized with acetone, washed with PBS, and then blocked with 10% normal goat serum for 30 min at room temperature. The cells were stained at 4 °C overnight with monoclonal anti-α-tubulin conjugate (Sigma-Aldrich) and nuclei were stained with 4′,6-diamidino-2-phenylindole (DAPI). For measurement of the distance from the nucleus, the MitoTracker signal was divided into rings of equal area from the border of the nucleus. The signal for mitochondria was measured at the average distance within each area and these values were plotted. The cell sizes were measured in μm using LSM 510 Meta, Carl Zeiss software.

For confocal staining of LC3 or BCL2, cells were plated in eight-well chamber slides, washed with PBS, fixed with methanol (10 min), permeabilized with acetone (1 min), washed again with PBS, and then blocked with 10% normal goat serum for 30 min. The cells were stained at 4 °C overnight with anti-LC3 (Novus Biologicals, Littleton, CO, USA) or anti-BCL2 (Santa Cruz Biotechnology, Santa Cruz, CA, USA) antibodies. The primary antibody was detected with phycoerythrin-conjugated anti-rabbit IgG secondary antibody for 2 h at room temperature, and the nuclei were stained with DAPI. The stained cells were analysed using a confocal microscope (LSM 510 Meta; Zeiss). The expression of LC3 or BCL2 was estimated by comparing the mean fluorescence intensity using LSM 510 Meta, Carl Zeiss software.

### Oxygen consumption rate (OCR)

The XF24 Extracellular Flux Analyzer (Seahorse Bioscience, Chicopee, MA, USA) was used to measure the OCR. OA and RA FLSs were plated at 1.5 × 10^4^ cells per well in XF24 well culture microplates. One day later, the cell culture medium was removed and the cells were cultured for 24 h in 250 μl/well of 0.1% ITSA–DMEM medium in 96-well plates in the presence or absence of IL-17 (10 ng/ml) or the supernatant collected from Th17 cells or Th17 cells co-cultured with FLS. The culture medium was then removed, and the cells were washed and cultured in XF assay medium supplemented with 1 mM sodium pyruvate, 2.5 mM glucose and 4 mM GlutaMax in a non-CO_2_ incubator. Mitochondrial electron transport was assessed through sequential injections of 4 μM oligomycin, 3 μM carbonyl cyanide 4-(trifluoromethoxy) phenylhydrazone (FCCP) and 2 μM rotenone/2 μM antimycin A. Basal respiration was calculated as baseline OCR—rotenone/antimycin A OCR; ATP-linked respiration as basal respiration—oligomycin OCR; maximum respiration rate as FCCP OCR—rotenone/antimycin A OCR; and reserve capacity as maximal respiration—basal respiration.

### Western blotting

The protein level of oxidative phosphorylation (OxPhos) complexes (I: 36 kDa/1621 bp, II: 70 kDa/2124 bp, III: 49.5 kDa/2226 bp, IV: 17 kDa/9219 bp and V: 53 kDa/471 bp; Invitrogen, Carlsbad, CA, USA), LC3 (I: 19 kDa and II: 17 kDa, Novus) and β-actin (Santa Cruz Biotechnology) was measured using a western blot system (SNAP i.d. Protein Detection System, Merck Millipore, Danvers, MD, USA). Cells were incubated in 100 mm dishes with 3-MA (1 mM) in the presence or absence of IL-17 (10 ng/ml) for 12 h, and mitochondrial and whole-cell lysates were prepared. The protein concentration was measured using the Bradford method (Bio-Rad, Hercules, CA, USA), and samples were separated on a 4–12% sodium dodecyl sulfate polyacrylamide gel and transferred to a nitrocellulose membrane (Amersham Pharmacia, Uppsala, Sweden). The primary antibodies to LC3, OxPhos complexes and β-actin were diluted in 0.1% skim milk in Tris-buffered saline Tween-20 and incubated for 15 min at room temperature. The membrane was washed and incubated with horseradish peroxidase-conjugated secondary antibody for 10 min at room temperature. Band density was estimated by image capture densitometry.

### Mitochondrial fractionation

RA FLSs were incubated in the presence or absence of IL-17 (10 ng/ml) for 12 h, and mitochondrial lysates were prepared. In brief, mitochondrial cell lysates were prepared from about 1 × 10^5^ cells by homogenization in mitochondria fractionation buffer with 0.02% digitonin. The lysates were centrifuged at 900 g for 2 min at 4 °C, and the supernatants were discarded. STM buffer: 250 mM sucrose, 50 mM Tris-HCl and 5 mM MaCl_2_ (STM) buffer was added to the pellets and pellets were centrifuged at 500 g for 15 min at 4 °C. The supernatants were discarded, STM buffer was added again to the pellets, and the pellets were centrifuged at 1000 g for 15 min at 4 °C. STM supplemented with 1% NP-40 buffer was added to the pellets and the pellets were centrifuged at 1000 g for 5 min at 4 °C. The resulting supernatants were used as the mitochondrial fraction.

### Analysis of gene expression by reverse transcription–quantitative real-time PCR (RT-qPCR)

Messenger RNA (mRNA) was extracted using TRIzol (Molecular Research Center, Cincinnati, OH, USA). cDNA was synthesized using the Superscript Reverse Transcription system (Takara, Shiga, Japan), then qPCR was performed using LightCycler FastStart DNA Master SYBR green I (Takara) following the manufacturer's instructions. All expression values were normalized to that of GAPDH mRNA. The primer sequences are described in Table 1.

### Cell proliferation analysis

Cell proliferation was analysed using a CCK-8 kit (Dojindo Laboratories, Kumamoto, Japan) according to the manufacturer's instructions. Briefly, CCK-8 is reduced by dehydrogenases in cells to yield an orange-coloured product (formazan).^46^ The amount of formazan dye generated by the dehydrogenases is directly proportional to the number of living cells. RA FLSs (5 × 10^3^ cells/well) were cultured in 200 μl of 0.1% ITSA–DMEM medium in a 96-well plate with SNP (1 mM) or 3-MA (1 mM) in the presence or absence of IL-17 (10 ng/ml) for 12 h. CCK-8 solution was added to each well, and the cells were incubated for 4 h. Absorbance was measured at 450 nm on a microplate reader.

### Annexin V and propidium iodide staining

RA FLSs were harvested, washed with PBS, and stained with fluorescein isothiocyanate-conjugated Annexin V (BD Biosciences) and propidium iodide (BD Biosciences) for 10 min at room temperature. The cells were subjected to flow cytometric analysis on a FACSCalibur instrument (BD Biosciences).

### Statistical analysis

The results are expressed as mean±standard deviation (S.D.) or mean±standard error of the mean (S.E.M.). The data were analysed with Student's *t-*test or the Mann–Whitney *U* test using Prism 5 software (GraphPad, La Jolla, CA, USA). *P*<0.05 (two-tailed) was considered significant.

## Figures and Tables

**Table 1:** Primers used for real-time PCR

*Human genes*	*Forward*	*Reverse*
*NDUFB5*	GCC ATG AGT TTG TTG CGG C	CCT TCG GAA AGC CAC GAG T
*NDUFS3*	TGC TGT CTC TGT GTT CAA GG	CTC GAA GCC ATA ATC TGT CAG G
*UQCRB*	GGA TGT TTC GCA TTA AGA GGG C	TGG TCC ACT GCT CTT TAG GC
*CYCS*	TGG GCC AAA TCT CCA TGG TC	ATT GGC GGC TGT GTA AGA GT
*COX5B*	AGT CCC CTC CAT CTC CAA CA	CTG GGG CAC CAG CTT GTA AT
*COX7B*	AGC CAC CAG AAA CGT ACA CC	TAA CTC TGC CAA CAG GGG AC
*ATP5O*	GAG AGG TTC TCT CCC CTC ACT	CAA GGT ACC TCT CCG CGA TG
*ATG5*	GAC CTT CTG CAC TGT CCA TCT	GCA ATC CCA TCC AGA GTT GC
*LC3*	CCG CAC CTT CGA ACA AAG AG	AAG CTG CTT CTC ACC CTT GT
*BCL2*	GGG GAG GAT TGT GGC CTT C	CAG GGC GAT GTT GTC CAC
*CASPASE 1*	TTT CCG CAA GGT TCG ATT TTC A	GGC ATC TGC GCT CTA CCA TC
*CASPASE 3*	ACA TGG CGT GTC ATA AAA TAC C	CAC AAA GCG ACT GGA TGA AC
*GAPDH*	TGC CAA ATA TGA TGA CAT CA	GGA GTG GGT GTC GGT GTT G
